# A Single Amino Acid in the HA of pH1N1 2009 Influenza Virus Affects Cell Tropism in Human Airway Epithelium, but Not Transmission in Ferrets

**DOI:** 10.1371/journal.pone.0025755

**Published:** 2011-10-05

**Authors:** Neeltje van Doremalen, Holly Shelton, Kim L. Roberts, Ian M. Jones, Ray J. Pickles, Catherine I. Thompson, Wendy S. Barclay

**Affiliations:** 1 Department of Virology, Division of Infectious Disease, St. Mary's Campus, Imperial College, London, United Kingdom; 2 Virus Reference Department, Health Protection Agency Microbiology Services Colindale, London, United Kingdom; 3 School of Biological Sciences, University of Reading, Reading, United Kingdom; 4 Cystic Fibrosis/Pulmonary Research and Treatment Center, Department of Microbiology and Immunology, University of North Carolina at Chapel Hill, Chapel Hill, North Carolina, United States of America; Johns Hopkins University - Bloomberg School of Public Health, United States of America

## Abstract

The first pandemic of the 21^st^ century, pandemic H1N1 2009 (pH1N1 2009), emerged from a swine-origin source. Although human infections with swine-origin influenza have been reported previously, none went on to cause a pandemic or indeed any sustained human transmission. In previous pandemics, specific residues in the receptor binding site of the haemagglutinin (HA) protein of influenza have been associated with the ability of the virus to transmit between humans. In the present study we investigated the effect of residue 227 in HA on cell tropism and transmission of pH1N1 2009. In pH1N1 2009 and recent seasonal H1N1 viruses this residue is glutamic acid, whereas in swine influenza it is alanine. Using human airway epithelium, we show a differential cell tropism of pH1N1 2009 compared to pH1N1 2009 E227A and swine influenza suggesting this residue may alter the sialic acid conformer binding preference of the HA. Furthermore, both pH1N1 2009 E227A and swine influenza multi-cycle viral growth was found to be attenuated in comparison to pH1N1 2009 in human airway epithelium. However this altered tropism and viral growth in human airway epithelium did not abrogate respiratory droplet transmission of pH1N1 2009 E227A in ferrets. Thus, acquisition of E at residue 227 was not solely responsible for the ability of pH1N1 2009 to transmit between humans.

## Introduction

On April 29, 2009 the Centre for Disease Control and Prevention (CDC) reported two epidemiologically unrelated cases of swine-origin influenza infection in California [1]. In the following weeks this virus spread throughout the world, causing the World Health Organisation (WHO) to declare a pandemic on June 11, 2009. By the time the pandemic was declared over in August 2010, two pandemic waves had been documented in the Northern hemisphere and the virus had spread to more than 214 countries, causing over 18,000 confirmed influenza-related deaths [2].

Whole genome sequencing of the newly emerged pandemic H1N1 2009 (pH1N1 2009) virus revealed a swine-origin background [3], [4]. Interestingly, pH1N1 2009 contained a gene segment constellation not found in previous swine surveillance samples. The HA and NS genes were derived from the classical H1N1 swine influenza virus lineage, the NA and M genes originated from the Eurasian H1N1 swine influenza lineage, and the polymerase and NP segments were from the triple-reassortment influenza viruses (TRIG) [3], that have circulated in North American pigs since a reassortment event in 1998 [5], [6]. In the last decade several H1 swine influenza viruses possessing the TRIG constellation were reported to have infected humans under exceptional circumstances, linked to direct contact of humans with pigs. However, these zoonotic events never progressed into a human pandemic, as consistent human-to-human transmission was not evidenced [7]. Recently it was demonstrated that the viruses involved in two such swine-origin dead-end human zoonoses were efficiently transmitted between ferrets in direct contact, but, in contrast to the pH1N1 2009 virus, transmission via respiratory droplets did not occur [8]. This implies that the H1/TRIG swine influenza viruses responsible for the sporadic human zoonotic events before pH1N1 2009 were not able to cause a pandemic, since efficient human to human transmission, which includes respiratory droplet transmission, is a prerequisite of a pandemic virus.

Historically, changes in the receptor binding protein of influenza, HA, have been implicated in the initiation of a pandemic. Wild aquatic birds form the natural reservoir of influenza virus and here the virus causes a gastrointestinal infection. In contrast, human influenza viruses usually cause an upper respiratory tract infection. The contrasting sites of infection are attributed to differences in receptor binding preferences; whereas the host receptor for avian influenza is α2,3-linked sialic acid found abundantly in the avian gut, human-adapted influenza viruses bind preferentially to α2,6-linked sialic acid expressed at the apical surface of the airway epithelia [9], [10], [11], [12], [13]. In order to overcome the species barrier and cause a pandemic, it is generally believed that one of the properties that avian influenza viruses must change is their receptor binding preference, from α-2,3 to α2,6-linked sialic acid.

In 1918, a H1N1 virus caused the first well-documented influenza pandemic of the 20^th^ century. Two mutations in the receptor binding site (RBS) of the H1 HA are thought to have played a vital role in enabling the transition from birds to man; residue 190 was an aspartic acid (D) rather than glutamic acid (E) which is commonly found in bird viruses, and residue 225 was aspartic acid (D) rather than glycine (G). These residues significantly affect the sialic acid preference of the H1 HA protein [14] and were experimentally shown to be required for virus transmission between ferrets [15]. Similarly, for the influenza H3N2 subtype, whose HA gene was introduced from an avian source to cause a pandemic in 1968, two other residue changes in the HA RBS have been linked to sialic acid preference, namely Q226L and G228S [9], [16], [17], [18], [19]. Interestingly the H1 HA of the classical swine viruses circulating at the turn of the 21^st^ century, from which pH1N1 2009 originates, already contained the human-like amino acids 190D and 225D but these viruses did not transmit between humans. It is therefore possible that other amino acid changes, unique to the HA of the pH1N1 2009, played a crucial role in the ability of the virus to cause a pandemic.

Glycan array studies comparing the carbohydrate specificity of the HA of swine-origin viruses involved in dead-end zoonoses to pH1N1 2009 HAs failed to identify an obvious ligand difference [20], [21], [22]. The glycans on these arrays are synthetic and might not represent the glycans expressed in the upper respiratory tract, which would explain why no difference was found.

Comparison of the HA amino acid sequence from seasonal H1N1, pH1N1 2009, swine-origin dead-end zoonoses and classic swine influenza viruses described in this paper identified residue 227 as a candidate amino acid that might affect receptor binding. Residue 227 is localised within the RBS and is commonly alanine (A) in swine influenza viruses but glutamic acid (E) in human seasonal and pH1N1. A recent study investigated the effect of residue 227 on the binding of H1 HA, expressed in HEK293S GnT1 cells, to a glycan array, and in contrast with the other two studies [20], [21] reported that wild type pH1N1 2009 HA did not bind strongly to any sialic acids. However, the 227A back-mutation resulted in increased α2,6-linked sialic acid binding [23].

Several studies have focused on the cell tropism of avian and human influenza viruses in mammalian airways. Differences have been found between the binding of avian and human viruses to cells in the upper respiratory tract of humans, pigs or ferrets [24], [25], [26]. Human airway epithelium (HAE) cultures are a favoured model as they represent differentiated cells normally found in the respiratory tract and contain naturally occurring glycans, in contrast to the previously discussed glycan arrays. In HAE cultures, avian influenza viruses exclusively target ciliated cells, and human influenza viruses bind preferentially to non-ciliated cells with some binding to ciliated cell types also seen [27], [28], [29], [30]. This difference is thought to be caused by the differential distribution of sialic acid on HAE; whereas ciliated cells express α2,3-linked sialic acids in addition to α2,6-linked sialic acid, non-ciliated cells are found to only express α2,6-linked sialic acids [31], [32].

In the present study, we investigated the effect of amino acid 227 in the HA of an isolate of pH1N1 2009, A/England/195/09, on cell tropism and multi-cycle growth in HAE cultures and on virus transmission via respiratory droplets in ferrets. Although amino acid 227 had a ‘humanizing’ effect on cell tropism and growth of pH1N1, a significant difference in transmission efficiency was not found.

## Results

### Comparison of amino acid residues 219 and 227 in HA protein of H1N1 viruses

**Figure 1:**
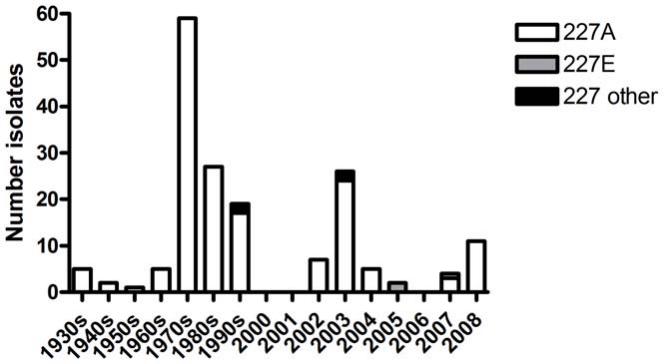
Distribution of amino acids at position 227 in swine H1 Has. Presence of alanine, glutamic acid and other amino acids at position 227 in 173 full-length HA sequences of pre 2009 North-American swine H1 influenza.

One hundred and seventy-three full-length HA sequences of classic swine influenza isolated before January 2009 in North America were compared to a randomly selected panel of 1397 sequences from seasonal H1N1, isolated between 1918 to 2008, and 1605 pH1N1 2009 as well as five published HA sequences of H1 dead-end zoonoses from swine to humans. The majority of recent seasonal H1 viruses and the 2009 pandemic viruses, both of which transmit efficiently between humans, contain glutamic acid (E) at residue 227. In contrast, human isolated swine-origin dead-end zoonoses and the classic swine influenza viruses contain alanine (A) at this position. The exceptions were early H1N1 viruses, such as the 1918 virus, which also had alanine at position 227 and yet were clearly transmissible between humans (*Table 1*). This contradiction may be explained by the additional influence of amino acid 219 which is alanine in 1918 H1 but threonine in the classical swine HAs. Cooperation between residue 219 and 227 has been suggested to influence the orientation of residue 186, which forms an interaction network with residues 187 and 189. This network then influences the orientation of residue 190 [33], which plays a vital role in receptor binding preference of H1N1 [14], [15]. Thus 1918 HA can tolerate the 227A and still bind efficiently to α2,6-linked sialic acid because it has a compensatory alanine at position 219. The majority of HA sequences of North American classical swine influenza (>95%) investigated contained alanine at position 227 (*Figure 1*) combined with threonine at position 219 potentially disrupting the interaction network around the receptor binding site. Another exception to our finding was a set of three swine influenza viruses isolated in 2005 and 2007 that contained 227E. However, phylogenetic analysis of these isolates (data not shown) showed a clustering with swine influenza viruses from the δ-cluster, which is associated with two separate introductions of human H1N2 and H1N1 into the swine population [34]. Moreover, these viruses also contained the human-like amino acid lysine (K) at position 219 reinforcing the concept that they were human derived viruses. One swine-origin dead-end zoonosis virus HA (FJ986622), containing glutamic acid at position 227, also clustered with the δ-cluster and is therefore also hypothesised to represent a zoonotic event of human-origin H1N2 from swine to human. It is likely that the lack of transmission of viruses of the δ-cluster is due to the influence of viral genes other than HA.

**Table 1:** Glutamic acid at position 227 in H1N1 influenza virus is associated with human-to-human transmission.

Isolate name		Host	Human to human transmission	Residue at	
				219	227
A/SC/1/1918	Pandemic	Human	Yes	A	A
A/England/654/07	Seasonal	Human	Yes	K	E
A/England/26/08	Seasonal	Human	Yes	K	E
A/California/07/09	Pandemic	Human	Yes	I	E
A/England/195/09	Pandemic	Human	Yes	I	E
A/Ohio/01/07	Dead-end zoonosis	Swine/Human	No	T	A
A/Wisconsin/87/05	Dead-end zoonosis	Swine/Human	No	T	A
A/Swine/Alberta/56626/03	Classical swine	Swine	No	T	A
A/Swine/Ontario/23866/04	Classical swine	Swine	No	T	A

Sequence comparison of amino acid 227 and 219 (H3 numbering) of HA protein of H1N1 viruses, showing prototypic examples of seasonal H1N1, pH1N1 2009, classical swine influenza and dead-end zoonoses from swine to humans.

These data indicate that the HA of the 2009 pandemic virus is unique amongst swine influenza H1 HAs as a result of a human virus like amino acid at residue 227 in the RBS.

### Binding of recombinant H1 HA proteins of E195, E195 E227A and Ohio01 to HAE and ferret nasal turbinate sections

**Figure 2:**
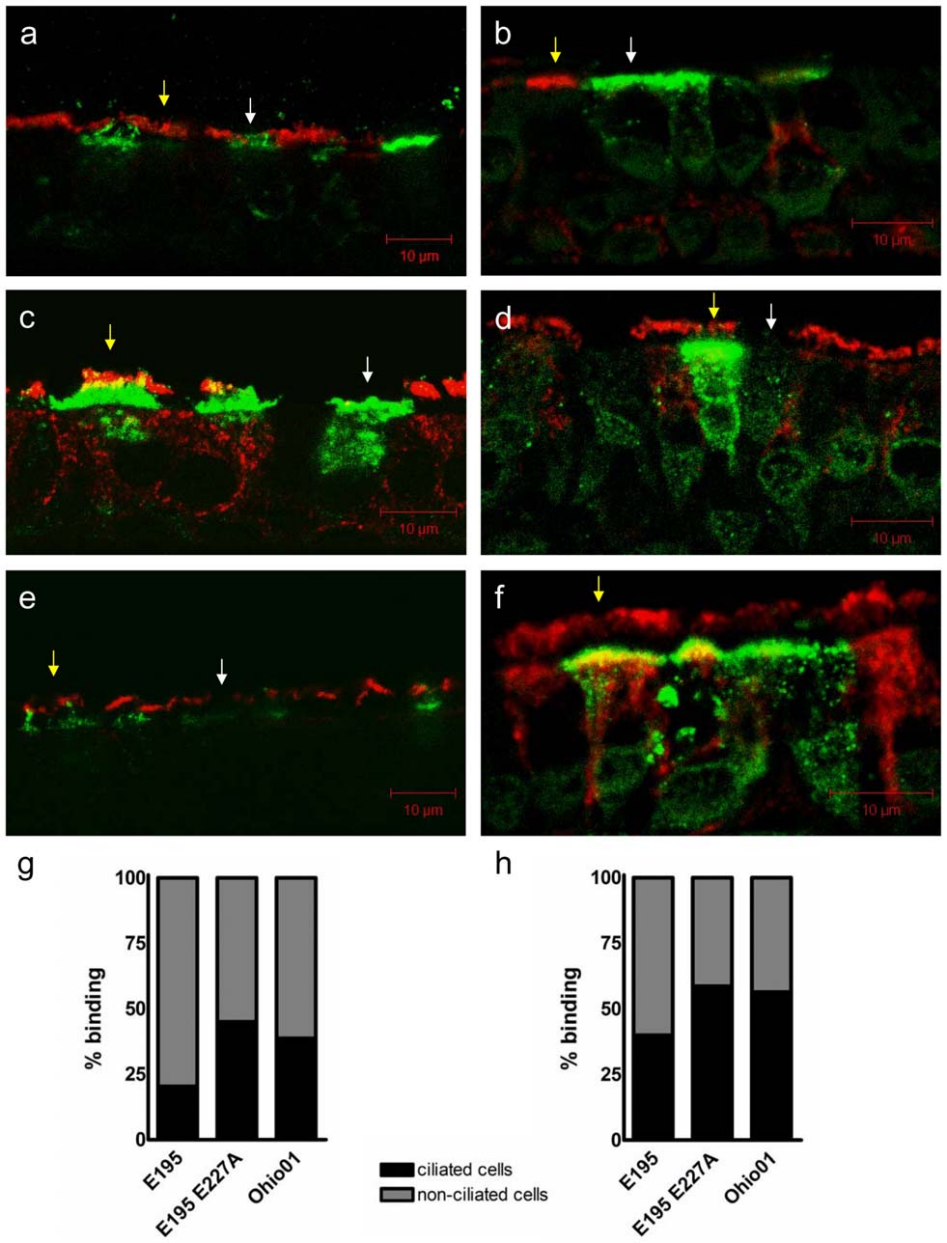
E195 shows a tropism preference for non-ciliated cells compared to E195 E227A and Ohio01 on HAE cultures. a/c/e) HAE culture sections were probed with HA-Fc proteins from human or swine H1N1 influenza virus strains. Ciliated cells were identified using anti-acetylated α-tubulin (red), and the HA-Fc proteins were visualized with anti-human Fc (green). Images are representative of multiple probed sections. Both non-ciliated cells (white arrow) and ciliated cells (yellow arrow) were present on human airway epithelium sections. b/d/f) HAE cultures were infected with MOI of 0.1 for 16 hrs. with isogenic viruses containing the HA genes of E195, E195 E227A or Ohio01. Ciliated cells were identified using anti-acetylated α-tubulin (red) and infected cells were detected with anti-NP antibody (green). Images are representative of multiple probed sections. Both non-ciliated cells (white arrow) and ciliated cells (yellow arrow) were present on human airway epithelium sections. g-h) Cell tropism was quantified by blind counting >200 cells per sample and expressed as the ratio of non-ciliated to ciliated cells bound or infected.

In order to investigate the effect of amino acid 227 on cell tropism of influenza virus, HA proteins of influenza viruses of interest were expressed in *Sf9* insect cells following fusion to the human Fc tag [28]. A/England/195/2009 (E195, GQ166661) was chosen as a prototypic pH1N1 2009 virus and a single residue variant, E227A, was created in addition to the parental sequence. The HA of a swine-origin dead-end zoonosis virus A/Ohio/01/2007 (Ohio01, FJ986620) was expressed similarly. Ohio01 is a swine influenza virus that infected a 36-year old man but did not transmit between humans. This virus contains internal gene segments from the TRIG constellation combined with the H1 HA from classical swine influenza. Ohio01 HA has the same primary protein sequence as the HA from A/Ohio/02/2007 used by Belser *et al*. [8] which did not transmit via respiratory droplets in the ferret model. These HAs both have alanine at residue 227. Formalin-fixed HAE sections containing a mixture of ciliated and non-ciliated cells were probed with each recombinant HA protein and processed as described [28]. Ciliated cells were identified using anti-acetylated α-tubulin (red) and cells bound by HA protein were detected with anti-human Fc antibody (green). The HA of E195 bound to non-ciliated cells (79.7%), as was previously observed for other HA proteins of human-adapted influenza viruses [28], [29], [30], [31] (*Figure 2a*). The single mutation at position 227 to alanine in HA broadened this binding profile, reducing the percentage of non-ciliated cells bound to 55% (*Figure 2c*) and the binding profile of the HA of Ohio01 showed a very similar pattern (61.3% non-ciliated cells bound) (*Figure 2e*). The relative non-ciliated/ciliated ratio was quantified by blind counting >200 cells per sample in 5-10 fields and was considerably higher for E195 HA (3.9) than for E195 E227A (1.2) or Ohio (1.6) HA proteins (*Figure 2g*).

We also tested the binding of each HA protein to fixed sections of uninfected ferret nasal turbinate (*Figure S1*). In contrast to the H5 HA from A/Vietnam/1194/04, which bound only weakly to the ferret turbinate tissue (*Figure S1d*), all three H1 HAs bound strongly to the ferret nasal turbinates (*Figure S1a,b,c*). Due to the variable nature of the *ex vivo* turbinate tissue it was not appropriate to quantify any differences in the tropism of the H1 HAs. However we did note a tendency of all three H1 HA proteins to bind to the apical surface of non-ciliated cell types, in contrast to the weak ciliated cell tropism demonstrated by the H5 HA.

### Infection of HAE by viruses with E195, E195 E227A and Ohio01 HA

Using reverse genetics [35], [36], isogenic viruses based on the E195 genetic backbone but containing HA genes of wild-type E195, E195 E227A or Ohio01 were created. HAE cultures were infected with each virus, using a multiplicity of infection (MOI) of 0.1 and fixed at 16 hours post infection (h.p.i). Ciliated cells were identified using anti-acetylated α-tubulin (red) and infected cells were detected with anti-NP antibody (green). Cell tropism was quantified by blind counting >200 cells per sample in 8-10 fields as before. The tropism preference of each virus mirrored the results obtained by recombinant HA protein binding on fixed HAE sections; wild-type E195 infected mostly non-ciliated cells (60.0%) (*Figure 2b*), the single mutation at position 227 to alanine broadened the infection preference to include more ciliated cells (41.4% non-ciliated cells infected, *Figure 2d*), which was similar to the cell tropism observed for virus with Ohio01 HA (43.6% non-ciliated cells infected, *Figure 2f* *)*. The non-ciliated/ciliated infected cell ratio was higher for E195 (1.5) compared to viruses with either E195 E227A (0.7) or Ohio01 (0.8) HAs (*Figure 2h*).

### Replication of recombinant influenza viruses with HA genes of E195, E195 E227A and Ohio01 in MDCK cells and HAE

**Figure 3:**
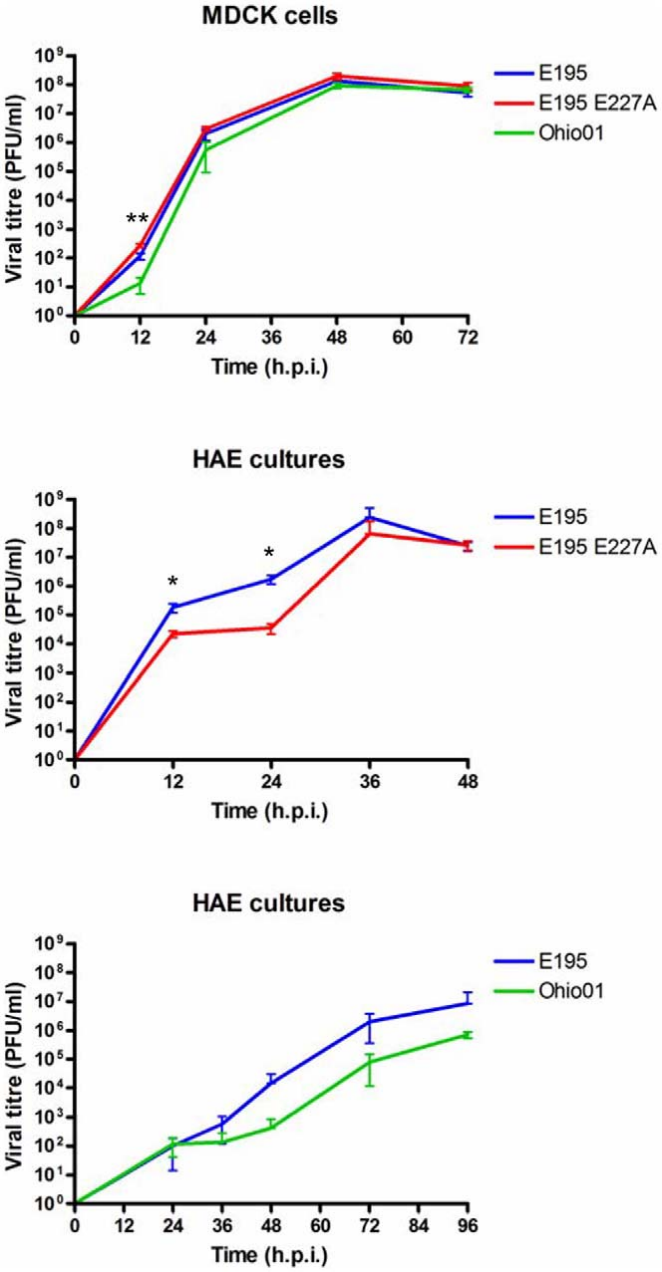
E195 E227A and Ohio01 are attenuated in HAE cultures. Comparison of multi-cycle virus growth in MDCK cells or HAE cultures inoculated at MOI of 0.001 with isogenic viruses based on the E195 genetic backbone but with different HA proteins; either E195, E195 E227A or Ohio01 HA. Viral titres were determined by standard plaque assay on MDCK cells. Data shown represent the mean titre and standard deviation of 3 cultures. Statistical differences between wild type E195 and E195 E227A or Ohio01 were determined using one-way ANOVA. *  =  p<0.05, **  =  p<0.01.

Based on the differences in cell tropism between E195 and both E195 E227A and Ohio01 in HAE, it was hypothesized that E195 E227A as well as Ohio01 would show attenuated replication in HAE cultures. E195 and the 227A mutant viruses grew with similar kinetics and to similar titres at 33°C in MDCK cells, the virus with Ohio01 HA was slightly attenuated (*Figure 3* *)*. However, in HAE cultures at 33°C, E195 E227A was significantly attenuated at 12 and 24 h.p.i. compared to E195 in HAE (p<0.05, *Figure 3*). In addition Ohio01 grew to lower viral titres than E195 at 33°C in HAE at each time point after 36 h.p.i., although the difference was not statistically significant (*Figure 3*). Note that the culture of HAE cells used for this latter analysis yielded lower titres of virus, likely due to donor variation, and thus the experiment was continued for a longer time period than for comparison of the E227A mutant above.

### Transmission of E195 and E195 E227A in a ferret model

The ferret model is generally accepted as the most appropriate system to investigate human-to-human transmission of influenza viruses as the distribution of sialic acid in the respiratory tract of humans and ferrets is comparable and ferrets display clinical signs typical of the symptoms of human disease [32], [37], [38]. We measured both direct contact transmission, via the co-housing of an inoculated donor ferret with a naive contact sentinel ferret, and respiratory droplet (RD) transmission by placing a sentinel animal in an adjacent cage separated by a perforated plastic barrier.

**Figure 4:**
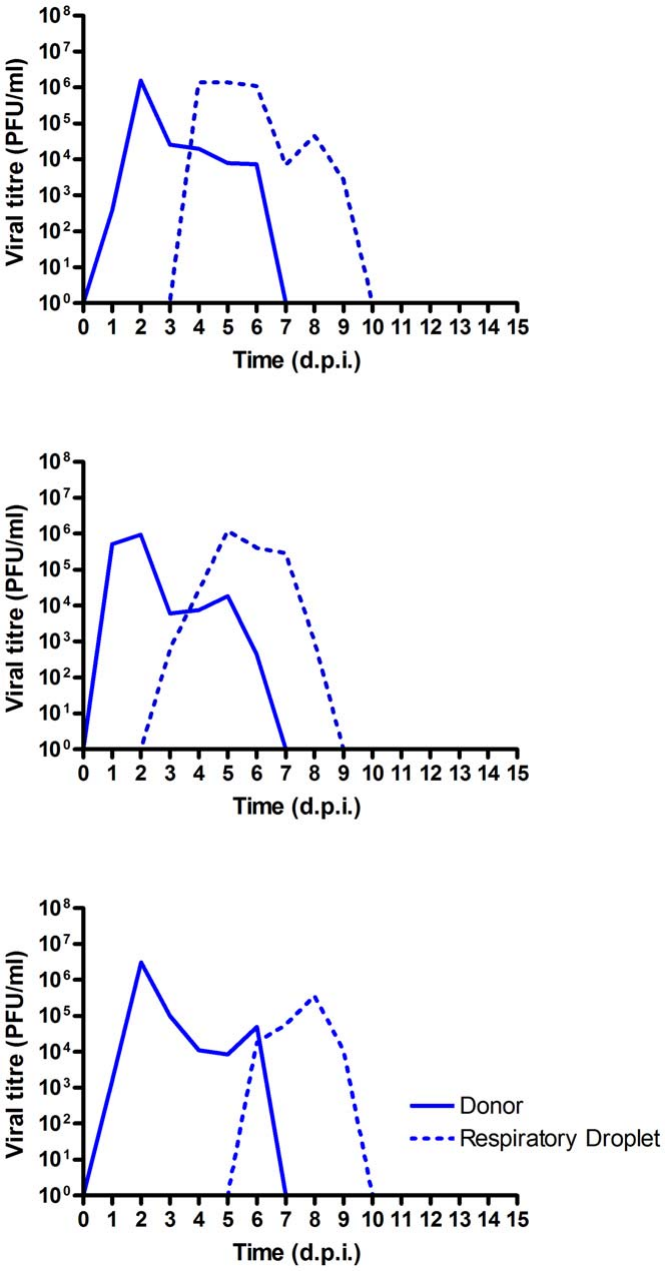
Transmission of wild type E195 in ferrets in respiratory droplet sentinels. Viral titres in nasal wash samples of ferret pairs, each pair contained one donor (solid lines) and one respiratory droplet (dotted lines). Donors were inoculated with 10^3^ PFU E195. Samples were taken daily and viral titres were determined by standard plaque assay on MDCK cells.

In preliminary studies we found that, like many other pH1N1 2009 virus studied in this manner [8], [33], [39], [40], E195 transmitted to all contact ferrets in a co-housed model and to 2 of 3 sentinel ferrets housed in adjacent cages by respiratory droplet transmission (data not shown). In the current study, we confirmed the respiratory droplet transmission of wild type E195 virus. Three ferrets were inoculated intranasally with 10^3^ PFU E195. Twenty-four hours post inoculation (p.i.) a RD sentinel was introduced to the cage adjacent to the donor animal and infectious viral shedding by both donors and respiratory sentinels was monitored by daily nasal washing and virus titration on MDCK cells. All donors inoculated with E195 became positive for infectious virus in nasal wash on day 1 p.i. Viral shedding in nasal wash was detected in all respiratory droplet sentinels starting on day 4 and 6. Peak E195 viral titres reached by respiratory droplet sentinels (average of 1.9×10^6^ PFU/ml) were comparable to peak viral titres reached by donors (average of 1.0×10^6^ PFU/ml) (*Table 2*) and viral shedding by sentinels was detected for 4 and 6 days (*Figure 4*) in line with inoculated donors that both shed infectious virus from the nose for 6 days.

**Table 2:** Summary of virus shedding from inoculated and respiratory droplet contact animals.

	Duration of shedding (days)		Peak titre (PFU/ml)		Area Under Curve (AUC) (Total PFU shed)	
	*E195*	*E195 E227A*	*E195*	*E195 E227A*	*E195*	*E195 E227A*
Donors	6	6	3.1×10^6^	4.0×10^6^	3.2×10^6^	4.2×10^6^
	6	6	1.6×10^6^	3.1×10^5^	1.6×10^6^	4.4×10^5^
	6	5	9.5×10^5^	1.4×10^6^	1.5×10^6^	1.4×10^6^
		5		3.5×10^7^		3.5×10^7^
**Average**	**6**	**5.5**	**1.9×10^6^** **(± 1.1×10^6^)**	**1×10^7^** **(± 1.7×10^7^)**	**2.1×10^6^** **(± 9.8×10^5^)**	**1×10^7^** **(± 1.7×10^7^)**
Respiratory	6	5	3.6×10^5^	1.7×10^7^	4.4×10^5^	1.8×10^7^
Droplets	6	5	1.4×10^6^	5.5×10^6^	4×10^6^	6.5×10^6^
	4	2	1.2×10^6^	1.4×10^4^	1.9×10^6^	2.3×10^4^
		2		3.8×10^3^		3.9×10^3^
**Average**	**5.3**	**3.5**	**9.8×10^5^** **(± 5.5×10^5^)**	**5.6×10^6^** **(± 8 x10^6^)**	**2.1×10^6^** **(± 1.8×10^6^)**	**6×10^6^** **(± 8×10^6^)**

Duration of shedding, peak titre and AUC were compared between ferrets directly inoculated or infected by respiratory droplet contact with wild type E195 or E195 E227A. Averages are displayed in bold with standard deviation indicated in brackets. The shedding parameters from ferrets infected by each virus were not significantly different as determined by Student's t-test.

**Figure 5:**
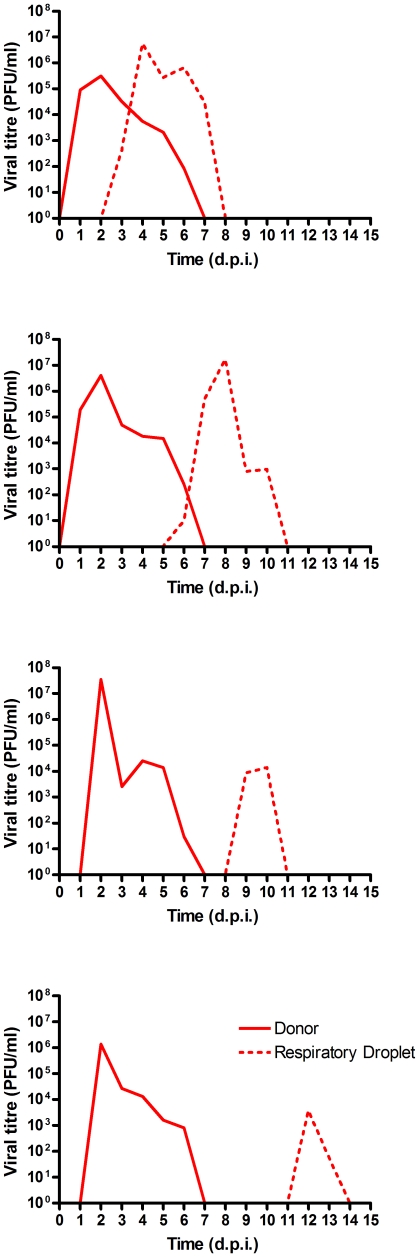
Transmission of E195 E227A in contact and respiratory droplet sentinels. Viral titres in nasal wash samples of ferret sets, each pair contained one donor (solid lines) and one respiratory droplet (dotted lines). Donors were inoculated with 10^4^ PFU E195 E227A. Samples were taken daily and viral titres were determined by standard plaque assay on MDCK cells.

Next we assessed the transmission of the E195 virus with the mutation in the HA receptor binding site at position 227 to alanine. Five donor ferrets were inoculated with 10^4^ PFU of E195 E227A via the intranasal route. A higher dose was used for this virus in order to assure robust infection of the donor animals given the modest attenuation of replication in the HAE cultures. Twenty-four hours p.i. two contact sentinel ferrets were introduced to two inoculated donors to assess contact transmission. A respiratory droplet sentinel was exposed to each of four of the inoculated donors in adjacent cages to determine if efficient respiratory droplet transmission was achieved by this virus. All of the donors inoculated with E195 E227A became positive for infectious virus in nasal wash on day 1 (2/4) or 2 (2/4) p.i (*Figure 5*). Viral titres shed were not significantly different than those shed from ferrets infected with wild type E195 virus (mean peak titre of mutant virus shed was 1.0×10^7^ PFU/ml, *Table 2*), and shedding duration (5–6 days) was also similar to that of the wild type E195 virus. Both contact sentinels were robustly infected (average peak shedding of 1.8×10^5^ PFU/ml) with no difference in duration (6 days in both contacts) in comparison to the inoculated donors of both the wild type E195 and E195 227A viruses.

Infectious virus was detected in at least two consecutive daily nasal washes for all four respiratory droplet sentinels (4/4). Virus shedding in nasal washes of the respiratory droplet sentinels with E195 E227A occurred at a range of times after first exposure and to various titres. Two respiratory sentinel animals shed robust virus titres (1.7×10^7^ and 5.5×10^6^ PFU/ml) with duration of 5 days comparable to wild type E195 respiratory droplet transmission. The other two respiratory sentinel animals showed a reduced duration of shedding (2 days) and considerably lower peak viral titre (1.4×10^4^ and 3.8×10^3^ PFU/ml) (*Figure 5*).

After transmission had occurred with E195 E227A, the HA genes of influenza viruses shed by donor and respiratory droplet sentinel animals were sequenced. Although most ferrets (7/8) shed E195 E227A, all five sequenced plaques of influenza virus shed by one respiratory sentinel were found to contain E195 A227T. No other mutations were found.

## Discussion

The emergence of the three influenza pandemics of the 20^th^ century has been associated with mutations in the HA protein that changed their receptor binding preference from α2,3-linked sialic acid to α2,6-linked sialic acid [14], [15], [16], [17], [18], [19].

The virus causing the first influenza pandemic of the 21^st^ century originates from a H1 swine virus [3], [4]. Interestingly, several human infections with H1 swine influenza viruses have been reported before pH1N1 2009. Human-to-human transmission was suspected in only one of these cases, but in other cases was not detected and these events certainly did not support a chain of human transmission that could lead to a pandemic [7]. We hypothesised that changes in the HA sequence of pH1N1 2009 enabled the virus to transmit between humans and was one of the factors that initiated the latest influenza pandemic.

Sequence comparison of the HA receptor binding site of seasonal H1N1, pH1N1, human infections with swine H1 and other swine H1 viruses indicated a remarkable difference at amino acid position 227. Whereas >95% of the swine H1 influenza virus and human infections with dead-end H1 human zoonoses contained alanine at position 227 in the HA, no pH1N1 2009 viruses were found with 227A. Instead, pH1N1 2009 contained glutamic acid at this position, the same amino acid as found at position 227 in all seasonal human H1N1 isolates since 1978, suggesting this position might serve to optimize the interaction between virus and the human host at the point of entry.

As an indicator of how the biological receptor binding characteristics might be affected by mutation at residue 227, we compared the cell tropism in cultures of primary HAE of a pH1N1 2009 strain A/England/195/09 (E195) to the HA mutant E195 E227A and the dead end human zoonotic virus Ohio01 using both HA protein binding as well as infection with recombinant viruses that varied in their HA gene. Published work using the HAE culture model has indicated that α2,3-linked sialic acid is found exclusively on ciliated cells and consequently avian influenza viruses bind and infect these cells predominantly in contrast to human transmissible viruses that preferentially bind and infect non-ciliated cell types [27], [28], [29], [30], [31]. Both E195 E227A and Ohio01 were found to bind and infect more ciliated cells than wild type E195 virus indicating a shift in receptor preference towards more α2,3-linked sialic acid binding, but the differences were subtle. The relevance of HAE binding profiles have yet to be directly correlated with transmissibility of influenza viruses but the tendency of viruses that are unable to transmit between humans to infect only ciliated cells [28], [30] suggests an entry preference that could be the foundation of transmission. Therefore by infecting an increased proportion of ciliated cells and a decreased number of non-ciliated cells in the HAE system, the likelihood of transmission *in vivo* might decrease. An interesting example of the interconnectivity of cell tropism and transmission can be found in a set of H3N2 recombinant viruses mutated in their RBS. After mutation of the human strain A/Hong Kong/1/68 at residues 226 and 228, the infected non-ciliated/ciliated ratio was changed from approximately 3 to <0.05, a 60 fold change [30]. The same mutations completely abolished transmission of a similar H3N2 virus, A/Victoria/3/75, in the ferret model [41]. In the present study the change in ratio of non-ciliated to ciliated cell binding and infection was only decreased by approximately two fold following acquisition of the swine signature 227A in the HA. This more subtle switch did not change the transmissibility of these viruses in the ferret model used here, suggesting that more dramatic changes in sialic acid specificity than are conferred by the E227A mutation lie behind the acquisition or loss of a transmissible phenotype. Indeed, analysis of the binding of the H1 HA proteins to uninfected ferret nasal turbinates, a target tissue for transmission, indicated that the cell type bound was predominantly a non-ciliated cell type, similar to the cells observed to be bound by the 1918 H1 HA [42]. Using the ex vivo tissue, we did not detect any obvious differences between the tropism of H1 HAs that differ at residue 227, but we emphasize that this type of biological sample can be highly variable which is why we used the more uniform airway cultures for quantitation of tropism differences. Maines et al. suggested that residue 227, which is found in the RBS of HA, in combination with residue 219 stabilizes the orientation of amino acid 190 [33], [43] which, in turn, is important in the receptor binding preference of HA [15]. They proposed that the combination of 227E with 219I found in the HA of pH1N1 2009 viruses destabilises this network and this may explain the lower α2,6-linked sialic acid binding of pH1N1 2009 HA as compared with that of 1918 HA [33]. Theoretical modelling of the pH1N1 HA led Soundararajan *et al*. to hypothesise that residue 227 stabilized the orientation of 222K [43], itself a component of a “lysine fence” that acts to anchor α2,6-linked glycans. This interaction and the importance of the lysine fence remain to be proven experimentally.

Jayaraman *et al*. corrected the interaction network between 227 and 219 in pH1N1 by changing residue 219 to the amino acid found in seasonal H1N1 in human isolates (lysine, K), thereby creating a theoretically more stable ionic network. This mutant showed both increased binding to sialic acid and increased respiratory droplet transmission in a ferret model. A second mutant, with the hydrophobic network corrected by changing three residues E227A, S186P and A189T, also showed increased α2,6-linked sialic acid binding compared to wild type, although no data on its transmissibility was yet presented [44]. Although these studies suggest mutational pathways by which pH1N1 2009 would increase its binding affinity to human receptors, it is interesting that these substitutions have not yet emerged during the virus transmission through human hosts [45].

De Vries et al. investigated the importance of residue 227 in binding sialic acid by comparing binding of fetuin, which contains α2,3-linked sialic acid and α2,6-linked sialic acid in an approximate 2∶1 ratio, to different HA proteins. HA proteins of swine influenza showed greater binding to fetuin compared to pH1N1 2009 HA. By creating chimeric proteins with regions of swine influenza HA or pH1N1 2009 HA, as well as by single-residue mutations it was found that introducing only E227A in pH1N1 increased fetuin binding and conversely introducing A227E in swine HA decreased fetuin binding. This clearly demonstrates that residue 227 alone is capable of changing the receptor binding profile of HA [23]. In the present study, an increased binding intensity to HAE was observed for E195 E227A HA (*Figure 2c*), which could be due to a correction of the hydrophobic network between 219 and 227 [33] similar to the increased binding of the same HA to fetuin reported by de Vries *et al*. [23].

A recent study showed that A/Ohio/02/07, a virus with an identical HA sequence to Ohio01, was able to transmit between co-housed ferrets, but not by respiratory droplet transmission [41]. The HA proteins of Ohio01/02 and E195 have an overall identity of 93.7%, differing in 36 amino acids, including residue 227, the only amino acid in the receptor binding site. We found that the multi-cycle replication kinetics in HAE of the E195 point mutant E227A and of the 7∶1 HA reassortant virus, Ohio01, were similar (*Figure 3*) suggesting that other differences in Ohio01 HA did not much affect virus replication in this system. Like the whole Ohio02 virus in the studies of Belser et al. (39), E195 E227A virus was shed robustly from donor animals and transmitted readily to 100% contact animals (data not shown). Unlike parental Ohio02 virus (39), the E195 E227A mutant virus also transmitted to all of the exposed sentinels through the respiratory droplet route. Since the Ohio01 and 02 viruses also differ from the 2009 pandemic virus in the origin of their NA and M gene segments, it is not clear whether lack of respiratory droplet transmission was due to the other genetic differences between HA of the Ohio 01/02 viruses and the pH1N1 HAs, or whether other genes in the Ohio02 virus limit its transmissibility.

Although the sample size was small (n = 4) it was observed that in two of the respiratory droplet sentinels, infection was delayed and peak titres were distinctively lower (*Figure 5*). In one of these animals the transmitted virus was found to contain a threonine at the site of manipulation, A227T. Five out of five plaques picked from the nasal wash of this sentinel contained the A227T mutation, yet this mutation was not present in any of five plaques picked from virus shed from the paired donor animal, suggesting that it had been selected for at the transmission bottleneck. Although threonine is not a charged residue like glutamic acid, they are both polar amino acids. Threonine at position 227 is occasionally observed in HA sequences of swine or human H1N1 influenza isolates, but not in human isolates of H5 or H3 influenza virus. Further studies are needed to investigate the significance of threonine at position 227.

Importantly, the other two animals who acquired E227A virus through the respiratory droplet route showed a shedding profile likely to support a chain of transmission. Therefore we conclude that the acquisition of the A227E mutation in a precursor of the 2009 pandemic virus is unlikely on its own to account for the emergence of human transmissibility phenotype.

## Materials and Methods

### Amino acid alignments

Swine influenza HA genes were selected from the NCBI Flu Database based on North-American origin, full length sequence and isolation before January 2009. 173 amino acid sequences were compared using Geneious 5.3.6 to five swine-origin dead-end zoonoses (GenBank accession numbers: FJ986618, FJ986619, FJ986620, FJ906621 and FJ986622) and a panel of 1397 seasonal and 1605 pH1N1 HA sequences representing the spectrum of human H1N1 influenza viruses.

### Cells

Madin-Darby Canine Kidney (MDCK) cells obtained from ATCC were maintained in DMEM (Gibco-Invitrogen, Inc.) supplemented with 10% fetal bovine serum (Biosera, Inc.), 1% penicillin/streptomycin (Sigma-Aldrich, Inc.) and 1% non-essential amino acids (Sigma-Aldrich, Inc.). Human airway epithelium cultures were either obtained from Cystic Fibrosis/Pulmonary Research and Treatment Center, Department of Microbiology and Immunology, University of North Carolina at Chapel Hill and were cultured as described previously [46], [47] or Epithelix Sàrl, Switzerland and cultured following manufacturer's protocol. Briefly, HAE cultures were grown in custom media with provision of an air-liquid interface for 4 to 6 weeks to form differentiated, polarized cultures that resemble in vivo pseudostratified mucociliary epithelium.

### Viruses

Recombinant viruses containing HA segments of wild-type A/England/195/2009 (H1N1), A/England/195/2009 E227A or A/Ohio/01/2007 combined with the remaining seven segments from wild-type A/England/195/2009 were generated from cloned cDNA in 293T and MDCK cell co-cultures via reverse genetics as previously described [36], [48].

### Expression of influenza HA protein in insect cells

Recombinant influenza HA proteins were generated and expressed as described previously [27]. Briefly, *Spodoptera frugiperda* cells (Sf9) were infected with recombinant baculovirus expressing H1-Fc receptor binding mutants. Supernatant was harvested and the soluble protein was concentrated using Vivaspin columns (Viva Science, Sartorius Group). Protein was stored at −80°C.

### Binding of influenza HA proteins to human or ferret airway epithelial culture sections

Paraffin embedded sections of cultured human airway epithelium (HAE) were obtained from UNC Cystic Fibrosis Center Core facility. Paraffin embedded section of ferret nasal turbinate were obtained following dissection of uninfected ferret airway. Binding of influenza HA proteins to HAE or fixed ferret tissue was determined as described previously [27]. Briefly, sections were deparaffinized and rehydrated. Non-specific binding was blocked using 3% bovine serum albumin before incubation with H1 proteins pre-complexed with goat anti-human IgG (Fc specific) antibody (Invitrogen) and rabbit anti-acetylated α-tubulin in 1% BSA. This was followed by incubation with donkey anti-goat FITC (Immunologicals Direct) and donkey anti-rabbit Alexafluor 647 (Invitrogen). Images were collected on a Zeiss Pascal LSM5 laser scanning microscope using Axioplan2 imaging software using a Plan-Apochromat 63×1.4 oil Ph3 lens. The binding of each mutant to the different cell types was quantified by blind counting at least five different fields of view containing a total of >200 cells.

### Infection of human airway epithelium with recombinant virus

HAE cultures were infected as described previously [46]. Briefly, HAE were washed with PBS to remove apical secretions. Virus inoculum was applied to the apical surface of HAE. After 1 hour of incubation at 33°C, HAE cultures were washed and incubated at 33°C for the duration of the experiment. Viral growth kinetics were determined by performing apical washes with 300 µl of serum-free DMEM for 30 min at 33°C. Washes were harvested and stored at −80°C prior to analysis. Viral titres in the apical washes were determined by standard plaque assay on MDCK cell monolayers as previously described [49].

### Infection of MDCKs with recombinant virus

6 well plates of MDCK cells were washed with PBS (Gibco). MDCK cells were infected with a MOI of 0.001 using virus diluted in serum-free DMEM (Gibco) for 1 hour at 33°C. The viral inoculum was removed and the MDCK cells were washed with PBS, after which two ml of serum-free DMEM supplemented with 1% penicillin/streptomycin, 1% non-essential amino acids and 1 µg/ml trypsin was added. MDCK cells were incubated at 33°C for the length of the experiment. 0.5 ml samples were removed from the infected 6 well plates (each virus was performed in triplicate) at each time point and 0.5 ml of serum-free DMEM supplemented with 1% penicillin/streptomycin, 1% non-essential amino acids and 1 µg/ml trypsin was replaced. Viral titres were determined by standard plaque assay on MDCK cell monolayers as previously described [49].

### Ethics Statement

All animal research described in this study was carried out under a UK Home Office License, PPL/70/6643.

### Animal studies

Animal studies were performed as described previously [41]. Twenty to 26 week old female ferrets weighing between 900–1250 g were used. After acclimatisation, sera were obtained and tested by microneutralisation (MN) assay for antibodies against A/England/195/2009 (H1N1). All ferrets were negative for influenza antibodies at the start of the experiments. Body weight of ferrets was measured daily. Strict procedures were followed to prevent cross-contamination between animals during all procedures. For inoculation, ferrets were lightly anaesthetised with ketamine (22 mg/kg) and xylazine (0.9 mg/kg) and inoculated intranasally with virus diluted in PBS, 0.1 ml per nostril. Twenty-four hours after inoculation of the donor ferrets, sentinels were introduced either as contacts or respiratory sentinels. All animals were daily nasal washed, consciously, by instilling 2 ml PBS into the nostrils and the expectorate was collected in modified 250 ml centrifuge tubes. BSA (0.3%), antibiotics (1%) and antifungal agent (1%) was added to nasal wash expectorate before viral titre was determined by standard plaque assay on MDCK cells monolayers as described previously [49]. The limit of virus detection in the plaque assays was 10 PFU/ml. Terminal blood samples were collected for sero-conversion determination.

## Supporting Information

Figure S1 **E195, E195 E227A and Ohio01 show a preference to non-ciliated cells on** ***ex-vivo*** **ferret nasal turbinate tissue.** *Ex vivo* ferret nasal turbinate tissue was probed with HA-Fc proteins from human (A/England/195/09 (a) and E227A mutant (b)), or swine (Ohio/01 (c)) H1N1 influenza virus strains or from avian H5N1 A/Vietnam/1194/04 virus (d). Ciliated cells were identified using anti-acetylated α-tubulin (red), and the HA-Fc proteins were visualized with anti-human Fc (green). Images are representative of multiple probed sections. Both non-ciliated cells (white arrow) and ciliated cells (yellow arrow) were present on *ex vivo* ferret nasal turbinate.
(TIF)
