# Supplementary figures and images for: A Single Amino Acid in the HA of pH1N1 2009 Influenza Virus Affects Cell Tropism in Human Airway Epithelium, but Not Transmission in Ferrets
Source: PLoS One. 2011 Oct 5;6(10):e25755. doi: 10.1371/journal.pone.0025755 (PMC3187803; doi:10.1371/journal.pone.0025755)

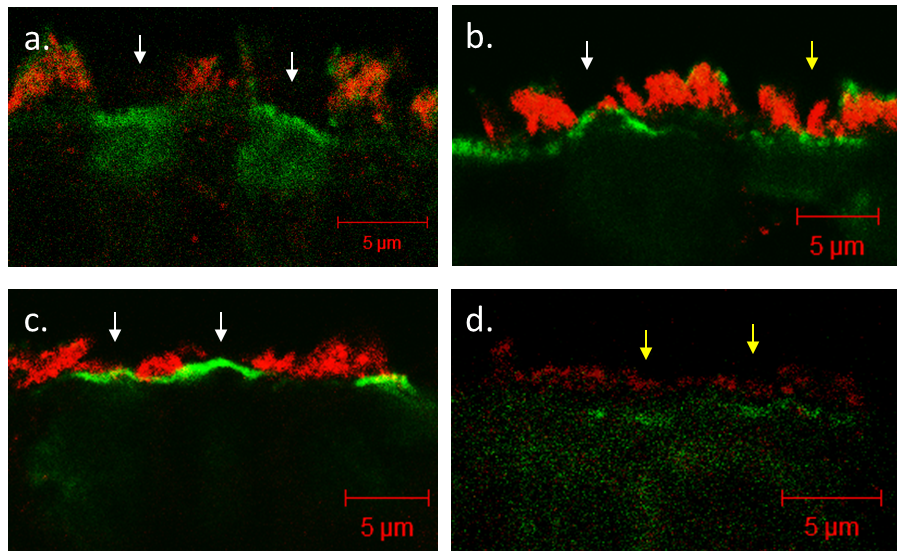

Supplement: Figure S1 — E195, E195 E227A and Ohio01 show a preference to non-ciliated cells on ex-vivo ferret nasal turbinate tissue. Ex vivo ferret nasal turbinate tissue was probed with HA-Fc proteins from human (A/England/195/09 (a) and E227A mutant (b)), or swine (Ohio/01 (c)) H1N1 influenza virus strains or from avian H5N1 A/Vietnam/1194/04 virus (d). Ciliated cells were identified using anti-acetylated α-tubulin (red), and the HA-Fc proteins were visualized with anti-human Fc (green). Images are representative of multiple probed sections. Both non-ciliated cells (white arrow) and ciliated cells (yellow arrow) were present on ex vivo ferret nasal turbinate. (TIF) [file pone.0025755.s001.tif]
